# Moderately Increased Albuminuria Is an Independent Risk Factor of Cardiovascular Events in the General Japanese Population under 75 Years of Age: The Watari Study

**DOI:** 10.1371/journal.pone.0123893

**Published:** 2015-04-07

**Authors:** Satoshi Konno, Masanori Munakata

**Affiliations:** 1 Division of Hypertension, Tohoku Rosai Hospital, Sendai, Japan; 2 Research Center for Life Style Related Disease, Tohoku Rosai Hospital, Sendai, Japan; University of Perugia, ITALY

## Abstract

**Background:**

Moderately increased albuminuria (formerly called microalbuminuria) is widely recognized as a predictor of cardiovascular disease. However, it is not clear whether this observation is applicable to the Asian population, as studies leading to this conclusion were conducted on Western populations. The aim of this study was to examine the hypothesis if moderately increased albuminuria could be an independent predictor of cardiovascular mortality and morbidity in the Japanese population.

**Methods and Results:**

The study population consisted of 3093 inhabitants of Watari, Miyagi Prefecture, who participated in an annual health check-up in 2009. We examined anthropometry, sitting blood pressure, fasting blood sample, and urine albumin-to-creatinine ratio (UACR). After baseline assessment, subjects were followed prospectively for up to 60 months. The incidence of major cardiovascular events (stroke, myocardial infarction, revascularization, and cardiovascular death) was determined based on death certificate records or medical claims sent to the National Health Insurance of Japan. Follow-up was discontinued for those who reached 75 years of age because they were moved to a different medical insurance system. We observed 57 cardiovascular events during a mean follow-up period of 47.8 months. The cumulative incidence rate for major cardiovascular events was significantly higher in patients with moderately increased albuminuria (UACR 30–299 mg/gCr) than in those with normoalbuminuria (UACR <30 mg/gCr) (6.4% vs. 2.2%, p = 0.0002 by log-rank test). Multivariate Cox proportional hazards analyses have revealed that moderately increased albuminuria is an independent predictor of cardiovascular events (HR 2.386, 95% CI: 1.120–4.390).

**Conclusions:**

Moderately increased albuminuria is an independent predictor of cardiovascular events in the general Japanese population under 75 years of age.

## Introduction

Moderately increased albuminuria, which was formerly called microalbuminuria and has been described as an early diagnostic marker of diabetic nephropathy [1], is now widely accepted as an independent predictor of cardiovascular disease in patients with diabetes [2], hypertension [3,4], and even in the general population [5–8]. Although a number of cohort studies [5–8] from Western countries and a meta-analysis [9] (including 1 Japanese trial) have reported the relationship between the presence of moderately increased albuminuria and future cardiovascular disease, this relationship has not been fully established in East Asian populations. Only a few studies have prospectively examined the relationship between urinary albumin excretion and cardiovascular mortality in the general Asian population [10, 11]. There are no data, however, showing that moderately increased albuminuria is predictive of cardiovascular morbidity in the general Asian population. We hypothesized that moderately increased albuminuria could be an independent predictor of cardiovascular mortality and morbidity in the Japanese population. To test this, we prospectively examined the association between moderately increased albuminuria and major cardiovascular events in a cohort study of a general Japanese population.

## Methods

### Study design and population

The Watari study is a community-based prospective cohort study of the general Japanese population conducted in the town of Watari, Miyagi Prefecture, Japan, that investigates the relationship between not only established risk factors but also moderately increased albuminuria and future cardiovascular disease. A detailed description of the study has been published elsewhere [12, 13]. The study protocol was approved by the ethics committee of Tohoku Rosai Hospital, and written informed consent was obtained from all participants.

A total of 3093 individuals from the general population (mean age 61.3 years; 40.2% men) who received an annual medical check-up in 2009 were enrolled. The cohort was closed at the end of July 2009 after the completion of baseline measurements and the participants were followed-up for major cardiovascular events (cardiovascular death, nonfatal myocardial infarction, angina pectoris requiring revascularization, and stroke) until the end of July 2014.

### Measurements

Information regarding past and current patient medical history and smoking status was obtained from a questionnaire. Trained nurses measured the height, body weight, and waist circumference of each participant. Body mass index (BMI) was calculated as weight (kg) divided by the square of the height (m^2^). After a 5-minute rest, sitting blood pressure was measured using a semiautomatic cuff-oscillometric sphygmomanometer (BX-10; Omron Colin, Kyoto, Japan). Blood samples were collected after an overnight fast and then analyzed for low- and high-density lipoprotein (LDL and HDL) cholesterol, triglycerides, HbA1c (NGSP), uric acid, and creatinine levels. HbA1c level was determined using HPLC (HLC723G7; Tosoh, Yamaguchi, Japan), and the level of the remaining biochemical markers was determined using a standard automatic analyzer (7700DD; Hitachi, Tokyo, Japan). The estimated glomerular filtration ratio (eGFR) was calculated using the formula provided by the Japanese Society of Nephrology [14]: eGFR (mL/min per 1.73 m^2^) = 194 × creatinine^-1.094^ × age^-0.287^ × (0.739 if female). Morning spot urine samples were also collected for the measurement of urinary albumin excretion. Urinary albumin and creatinine concentrations were determined using the turbidimetric method (Au600; Olympus, Tokyo, Japan) and enzymatic method, respectively. Urinary albumin excretion was expressed as the urine albumin-to-creatinine ratio (UACR).

### Outcomes

Death resulting from cardiovascular disease or a composite of first major cardiovascular events, such as nonfatal myocardial infarction, angina pectoris requiring revascularization, and stroke (intracerebral hemorrhage, subarachnoid hemorrhage, and cerebral infarction) were observed. We ascertained the cause of death from death certificate records. The incidence of cardiovascular events was examined from the electronic database of medical receipts sent to the National Health Insurance. It is mandatory for all Japanese residents to join the public health insurance system, which provides healthcare needs, including coverage of medical costs; this allowed us to examine a patient’s medical costs and their causative diseases using the receipts. All medical receipts indicating myocardial infarction, angina pectoris, intracerebral hemorrhage, subarachnoid hemorrhage, and cerebral infarction were recorded every month by trained municipal staff. The diseases indicated on the receipts are used for charging medical costs by medical facilities and does not necessarily indicate the definite diagnosis. To examine if the disease indicated a true event or not, the receipts were further reviewed by a trained physician who was blinded to the baseline data. The electronic database contains all the relevant information about medical treatment including prescribed drugs, devices used, operative procedures, etc. The treatment procedure was indicated in a chronological order, and a clinical summary was provided in cases of high-cost treatment. We examined if the disease indicated on the receipt was a true cardiovascular event based on a thorough review of the receipt.

To verify the validity of this approach, we studied the concordance rate between the medical receipts review and the gold standard method of reviewing the patient’s medical chart, using the medical record database of Tohoku Rosai Hospital. First, we selected all medical receipts from inpatients who had been hospitalized at Tohoku Rosai Hospital from the beginning of November 2013 to the end of March 2014 indicating the following diseases: myocardial infarction, angina pectoris, intracerebral hemorrhage, subarachnoid hemorrhage, and cerebral infarction. There were 50 cases who met the inclusion criteria: 31, angina pectoris; 5, unstable anginas; 4, acute myocardial infarctions; 4, old myocardial infarctions; 4, cerebral infarctions; 1, post-cerebral infarction case; and 1, post-cerebral bleeding case. We examined if the disease indicated on the respective patient’s receipt was a true event or not using a thorough examination of the medical receipts. Twelve out of the 31 angina pectoris cases were judged to be true events needing revascularization, and the remaining 19 angina pectoris cases and 4 old myocardial infarction cases only required diagnostic catheterization. Five unstable angina cases and 4 acute myocardial infarction cases were all true events that needed revascularization. Two of the 4 cerebral infarctions were hospitalizations for rehabilitation and the remaining 2 cases, 1 post-cerebral infarction and 1 post-cerebral hemorrhage, were judged to be hospitalization for observation.

The analysis was also done using the gold standard method by another physician unaware of the results of the medical receipts review. Consequently, 49 out of the 50 assessments coincided with those of the gold standard, and the concordance rate was 98.0%. The final case was not successfully determined by the medical receipts review because the patient was diagnosed with a cerebral infarction just after admission and was promptly transferred to a special hospital for thrombolytic therapy. In the setting of the Watari cohort study, however, information of multiple admissions of 1 patient was compiled in the report to the National Health Insurance and sent to the town office. We believe, therefore, that we have collected true events at a rate nearly identical to the gold standard method.

### Statistical analysis

**Fig 1:**
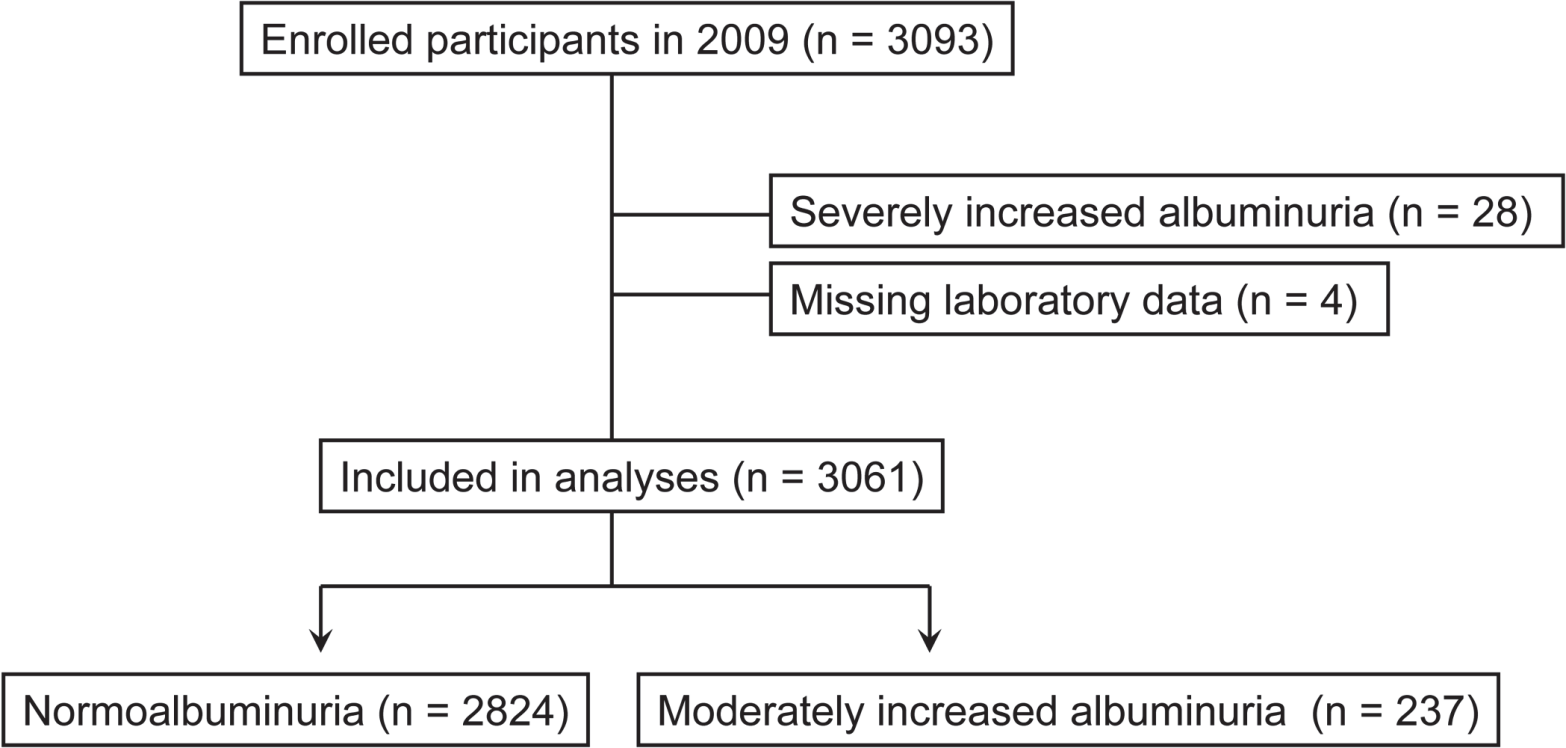
Flow chart of the selection of study participants.

According to the guidelines from the Japanese Society of Nephrology [15], normoalbuminuria is defined as a UACR of <30 mg/gCr, moderately increased albuminuria as 30–299 mg/gCr, and severely increased albuminuria (formerly called macroalbuminuria) as ≥300 mg/gCr. After excluding individuals with severely increased albuminuria at baseline (n = 28) and missing laboratory data (n = 4), 3061 participants were included in the final analysis (Fig 1).

Differences between participants with a baseline normoalbuminuria and moderately increased albuminuria were compared using the unpaired t-test or chi-square test. The Kaplan-Meier analysis with log-rank test was used to estimate cumulative event curves and to compare the normoalbuminuria and moderately increased albuminuria groups. Data were censored when participants were dead, suffered from predefined events, lost to follow-up, or were reaching the age of 75 years. We used Cox’s proportional hazards model with stepwise variable selection to estimate the hazard ratios (HR) and 95% confidence intervals (CI) for the composite outcome. Data were log-transformed in case of skewed distribution.

Hypertension was defined as systolic blood pressure ≥140 mmHg or diastolic blood pressure ≥90 mmHg, or the use of anti-hypertensive medications. Diabetes was defined as fasting blood glucose levels ≥126 mg/dL or HbA1c ≥6.5%, or the use of hypoglycemic medications. Dyslipidemia was defined as LDL-cholesterol levels ≥140 mg/dL or HDL-cholesterol levels <40 mg/dL or triglyceride levels ≥150 mg/dL, or use of lipid-lowering agents.

All statistical analyses were performed using JMP 9.0 for Windows (SAS Institute, Cary, NC, USA). Continuous variables were expressed as mean ± SD, and categorical variables as percentage. A p-value <0.05 was considered to be statistically significant.

## Results

At baseline, 237 participants (7.7%) demonstrated moderately increased albuminuria, and 2824 had normoalbuminuria (Table 1).

**Table 1:** Baseline characteristics of the study participants.

Variable	Total(n = 3061)	Normoalbuminuria(n = 2824)	Moderately increased albuminuria(n = 237)	p
Age, years	61.3 ± 11.4	60.9 ± 11.6	65.6 ± 7.7	<0.001
Men, %	40.1	39.2	49.8	0.001
Waist circumference, cm	83.9 ± 9.4	83.6 ± 9.3	86.7 ± 10.1	<0.001
Body mass index, kg/m^2^	23.2 ± 3.3	23.2 ± 3.3	24.3 ± 3.8	<0.001
Systolic BP, mmHg	131.4 ± 19.7	130.4 ± 19.1	143.6 ± 22.0	<0.001
Diastolic BP, mmHg	74.9 ± 11.3	74.3 ± 11.0	81.2 ± 12.4	<0.001
LDL-cholesterol level, mg/dL	123.4 ± 30.8	123.0 ± 30.4	127.3 ± 34.4	0.042
HDL-cholesterol level, mg/dL	62.9 ± 15.7	63.3 ± 15.7	57.9 ± 15.6	<0.001
Triglyceride level, mg/dL	107.3 ± 67.6	105.4 ± 65.8	129.9 ± 83.5	<0.001
HbA1c (NGSP), %	6.0 ± 0.6	5.9 ± 0.6	6.4 ± 1.1	<0.001
eGFR, mL/(min·1.73m^2^)	78.7 ± 15.6	78.8 ± 15.5	77.1 ± 17.1	0.113
Hypertension, %	45.6	43.4	72.2	<0.001
Diabetes, %	12.4	11.1	28.7	<0.001
Dyslipidemia, %	50.1	49.3	59.5	0.003
Smoking, %	13.4	12.8	20.3	0.001
UACR, mg/gCr	14.6 ± 24.5	9.3 ± 5.4	77.5 ± 55.8	<0.001

Data are presented as means ± standard deviation or percentages. p-value, unpaired t-test or chi-square test (normoalbuminuria group vs. Moderately increased albuminuria group). BP, blood pressure; LDL, low-density lipoprotein; HDL, high-density lipoprotein; HbA1c, glycated hemoglobin; NGSP, National Glycohemoglobin Standardization Program; eGFR, estimated glomerular filtration ratio; UACR, urine albumin-to-creatinine ratio.

Compared with the normoalbuminuria group, participants with moderately increased albuminuria were older, included more men and had higher systolic and diastolic blood pressures. Both glucose and lipid profiles were worse in the moderately increased albuminuria group. The frequency of smokers was higher in the moderately increased albuminuria group than in the normoalbuminuria group. Medications were more frequently prescribed in the moderately increased albuminuria group than in the normoalbuminuria group for hypertension (42.2% vs. 26.2%, p<0.001) and diabetes (11.0% vs. 4.3%, p<0.001), but similar for dyslipidemia (14.4% vs. 15.6%, p = 0.614).

During a mean follow-up period of 47.8 months, 57 participants developed major cardiovascular events (5 cardiovascular deaths, 6 acute myocardial infarctions, 11 angina pectoris requiring revascularization, and 35 strokes). During the maximum follow-up period of 60 months, 754 (24.6%) participants discontinued follow-up because they reached 75 years of age and transferred to a different medical insurance system catering to those ≥75 years of age. Another 530 participants (17.3%) withdrew from the study because of changes in residence or insurance provider. Thus, a total of 1284 participants were lost to follow-up; the final follow-up rate was 58.1%.

Compared with participants who were successfully followed-up, the lost to follow-up individuals had a significantly lower waist circumference, body mass index, diastolic blood pressure and LDL-cholesterol levels (S1 Table).

**Fig 2:**
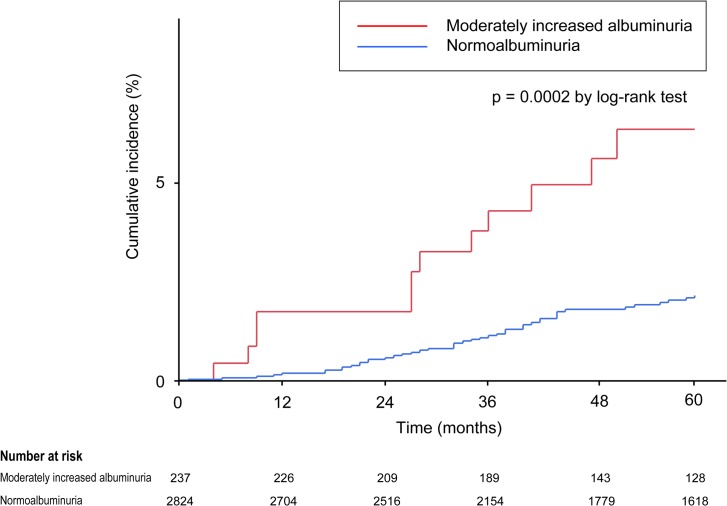
Kaplan–Meier curves for cumulative incidence of major cardiovascular events in normoalbuminuria and microalbuminuria groups.

The Kaplan-Meier analysis indicated that the cumulative incidence for major cardiovascular events was significantly higher in the moderately increased albuminuria group than in the normoalbuminuria group (6.4% vs. 2.2%, p = 0.0002 by log-rank test; Fig 2).

Univariate Cox regression analysis revealed that age, male gender, waist circumference, BMI, systolic and diastolic blood pressures, HDL-cholesterol, treatment for hypertension, and the presence of moderately increased albuminuria were associated with an increased risk of major cardiovascular events, as summarized in Table 2.

**Table 2:** Results of the univariate Cox proportional hazard analysis for major cardiovascular events.

Variable	Hazard ratio	95% CI	p
Age, per 1 year increase	1.071	1.029–1.123	<0.001
Men (vs. women)	2.653	1.557–4.655	<0.001
Waist circumference, per 1 cm increase	1.041	1.013–1.070	0.004
Body mass index, per 1 kg/m^2^ increase	1.085	1.007–1.165	0.033
Systolic BP, per 10 mmHg increase	1.202	1.058–1.359	0.005
Diastolic BP, per 10 mmHg increase	1.349	1.073–1.689	0.011
LDL-cholesterol level, per 10 mg/dL increase	1.032	0.948–1.118	0.461
HDL-cholesterol level, per 10 mg/dL increase	0.652	0.528–0.795	<0.001
Log-triglyceride, per 1 log increase	2.761	0.836–8.536	0.095
Log-UACR, per 1 log increase	3.059	1.628–5.443	<0.001
HbA1c (NGSP), per 1% increase	1.310	0.945–1.674	0.098
eGFR, mL/(min·1.73 m^2^)	1.000	0.983–1.017	0.966
Hypertension	1.703	1.009–2.927	0.046
Diabetes	1.839	0.929–3.361	0.078
Dyslipidemia	1.094	0.650–1.856	0.734
Smoking	1.407	0.671–2.667	0.345
Moderately increased albuminuria (categorical)	3.189	1.612–5.830	0.002

Abbreviations as shown in Table 1.

We performed a multivariate analysis using all significant covariates in the univariate analysis. After backward stepwise variable selection, age, male gender, lower HDL-cholesterol, and the presence of moderately increased albuminuria were identified as independent predictors of future cardiovascular events (Table 3).

**Table 3:** Results of the multivariate Cox proportional hazard analysis for major cardiovascular events.

Variable	HR	95% CI	p	HR	95% CI	p
Age, per 1 year increase	1.052	1.012–1.102	0.008	1.057	1.017–1.107	0.004
Men (vs. women)	2.034	1.171–3.632	0.011	1.949	1.124–3.476	0.017
HDL-cholesterol level, per 10 mg/dL increase	0.733	0.590–0.898	0.002	0.734	0.591–0.898	0.002
Moderately increased albuminuria (categorical)				2.386	1.120–4.390	0.015
Log-UACR, per 1 log increase	2.335	1.252–4.132	0.009			

Abbreviations as shown in Table 1.

Moderately increased albuminuria was associated with approximately a 2.4-fold higher risk of cardiovascular events than normoalbuminuria. The results were essentially similar if log-UACR was used instead of the presence of moderately increased albuminuria, as shown in Table 3.

## Discussion

In the present study, we have demonstrated that moderately increased albuminuria is an independent predictor of cardiovascular morbidity and mortality in the general Japanese population, despite relatively few cases of major cardiovascular events and a short follow-up period. Only two studies have examined the relationship between moderately increased albuminuria and cardiovascular mortality in the general Asian population [10, 11], and no studies have confirmed the significant relationship between moderately increased albuminuria and cardiovascular morbidity. Thus, this is the first report showing that moderately increased albuminuria can predict cardiovascular morbidity in the general Asian population.

We did not examine the relationship between moderately increased albuminuria and total mortality rates because the Great East Japan Earthquake and subsequent tsunami struck Watari on March 11, 2011, about 20 months after the initiation of this study. To date, 306 inhabitants of the town (nearly 1% of the total town population) have been listed as dead or missing owing to the disaster, which accounts for 45.9% of the total number of deaths in the town in 2011. In other words, the total mortality during this follow-up period does not seem to reflect the usual non-cardiovascular deaths of this town. The great disaster, however, did not appear to significantly alter the Kaplan-Meier incidence plot, as shown in Fig 2, suggesting that moderately increased albuminuria could be a robust predictor of cardiovascular events in the general Japanese population.

In a cohort study, the key to study quality is precisely and fully capturing measured events. Conventional approaches such as interviews or self-report questionnaires are often problematic in terms of cost or response rate. The World Health Organization: Multinational MONItoring of trends and determinants in CArdiovascular disease (WHO MONICA) project manual states that the examination of death certificates or hospital admission/discharge records are obligatory among many sources of information to identify cardiovascular events [16]. However, it is not easy to obtain all the hospital admission records, especially when a patient is hospitalized in a distant place. In this study, we collected data from National Health Insurance receipts as a source of identifying cardiovascular events. This approach offers a significant advantage over traditional methods in capturing cardiovascular events, because all the medical treatment information of all the study participants was sent to the local municipal government every month. This procedure enabled us to track the incidence of cardiovascular events during the follow-up period with considerable accuracy, even after the Great East Japan Earthquake, when a proportion of study participants temporarily evacuated from the affected region of Watari.

The present study did not include transient ischemic attack, cerebral infarction without thrombolytic therapy or anticoagulant therapy, or angina pectoris without revascularization as cardiovascular events to minimize the false positive cases. Moreover, the concordance rate between this approach and the final diagnosis recorded on the medical chart was nearly perfect in our validation study, indicating the sufficient validity of this new method of event evaluation.

We have previously reported in the same cohort that high-normal diastolic blood pressure, hypertriglyceridemia, and hyperglycemia are associated with an increased risk of future moderately increased albuminuria [13]. In conjunction with the present study, our data indicate that increases in blood pressure level, triglyceride level, and glucose concentration may contribute to the risk of cardiovascular events through the development of endothelial dysfunction.

Another interesting finding of the current study was that a reduced HDL-cholesterol level was also an independent predictor of cardiovascular events. These results suggest that a reduced HDL level is involved in the progression of atherosclerosis through different mechanisms, as indicated by moderately increased albuminuria. HDL elicits multi-anti-atherogenic effects through anti-oxidation and anti-inflammation, in addition to being a key mediator of reverse cholesterol transport [17]. These actions seem to be independent of renal endothelial damage as measured by urinary albumin excretion.

Although many studies conducted in Western countries have shown that a higher LDL level is associated with a greater risk of cardiovascular events [18–20], similar results were not confirmed in this study. There could be several reasons for this difference. First, the total number of events was small (n = 57) and may have less statistical power. Second, coronary artery disease was reported much less than stroke, the former being much more dependent on LDL level. Third, the risk of patients with hyper-LDL cholesterolemia might be attenuated by statin treatment because approximately 15% of the total population had received anti-dyslipidemia agents. Therefore, we need further follow-up studies to validate our findings.

This study has several limitations. First, the study participants were recruited at a medical check-up on a voluntary basis; therefore, they are not necessarily representative of the whole population of the region. Second, more than 20% of the participants were automatically classified as lost to follow-up because they turned 75 years of age and transferred to a different National Health Insurance coverage, which is currently managed by the prefectural government and hard to access. To improve the follow-up rate, we need an effort to receive a permission to access medical receipts of those older subjects. This process needs time and should be a future issue. Finally, the follow-up period of the present study was relatively short and the number of cardiovascular events was not large, which may have resulted in a reduced statistical power.

In conclusion, this study demonstrated for the first time that moderately increased albuminuria is an independent risk factor for cardiovascular events in the general Japanese population under 75 years of age. Further prospective studies with larger samples may be needed to confirm the predictive value of moderately increased albuminuria in cardiovascular morbidity in the Japanese population.

## Supporting Information

S1 Table Baseline characteristics of followed and not followed participants. Data are presented as means ± standard deviation or percentages. P-value, unpaired t-test or chi-square test (followed vs. not followed). Abbreviations as shown in Table 1.
(XLSX)
